# Pathogenic mechanisms of adult degenerative scoliosis

**DOI:** 10.3389/fcell.2026.1949453

**Published:** 2026-09-21

**Authors:** Hongqi Deng, Pengfei Ji, Xiaoling Wang, Yandi Cheng, Peng Wang, Wenjie Liu

**Affiliations:** 1 Department of Orthopedics, The Eighth Affiliated Hospital, Sun Yat-sen University, Shenzhen, China; 2 Guangdong Provincial Clinical Research Center for Orthopedic Diseases, The Eighth Affiliated Hospital, Sun Yat-sen University, Shenzhen, China; 3 Biological Laboratory of Hetao Cooperation Zone, The Eighth Affiliated Hospital of Sun Yat-sen University, Shenzhen, China

**Keywords:** adult degenerative scoliosis, asymmetric intervertebral disc degeneration, asymmetric paraspinal muscle degeneration, facet joint degeneration, spinal deformity

## Abstract

Adult degenerative scoliosis (ADS) is not merely a unidimensional sagittal decompensation, but rather a three-dimensional pathological process driven by asymmetric degeneration of intervertebral discs, endplates, facet joints, and paraspinal muscles, wherein coronal scoliosis, sagittal imbalance, and vertebral axial rotation mutually couple and amplify one another. Endplate tilting and nucleus pulposus displacement convert segmental mechanics to an asymmetric pattern, representing the initiating event; all three axes are reciprocally driven through facet joints and ligamentous tension, forming a self-amplifying progression loop; paraspinal muscles on the convex and concave sides exhibit opposing degenerative patterns that exacerbate instability; facet joint sagittalization transitions scoliosis from a compensable to a structurally fixed state; bone density loss and genetic susceptibility modulate individual progression rates. This review systematically synthesizes the above mechanisms and proposes a Three-Axis Reciprocal Coupling Pathogenic Model as a conceptual, hypothesis-generating framework, and discusses potential mechanism-informed intervention directions together with the limitations of the current evidence base. ADS is best understood as a systemic musculoskeletal syndrome involving multiple tissue systems, and this conceptualization may carry implications for early identification and precision intervention pending further validation.

## 1 Introduction

With accelerating global population aging, adult spinal deformity (ASD) has become one of the most prevalent spinal conditions among older adults. Influenced by variations in diagnostic criteria and population composition, reported prevalence rates range widely from 8% to 68% (Diebo et al., 2019; Kim et al., 2022). Adult degenerative scoliosis (ADS), also referred to as degenerative lumbar scoliosis (DLS), is an important ASD subtype defined as a newly developed scoliotic deformity with a coronal Cobb angle exceeding 10° arising after skeletal maturity (Aebi, 2005; York and Kim, 2017). A retrospective study of 2,973 individuals reported a primary degenerative scoliosis prevalence of approximately 8.85% in the general population aged 40 years and older, which increased with age; no significant sex difference was observed (Kebaish et al., 2011). Over the past decade, research on ADS pathogenesis, imaging assessment, and surgical strategies has grown substantially, and bibliometric analysis indicates the field entered a period of rapid expansion around 2010, with biomechanics and spino-pelvic sagittal parameters as current research focal points (Geng et al., 2024).

ADS differs fundamentally in pathological nature from purely sagittal spinal degenerative deformities. The latter primarily manifests as reduction or loss of physiological lumbar lordosis driven by anterior column height loss, essentially confined to the sagittal plane (Pritchett and Bortel, 1993). ADS, by contrast, is a genuine three-dimensional deformity in which coronal scoliosis, sagittal imbalance, and vertebral axial rotation evolve simultaneously in a coupled fashion (Geng et al., 2024; Pritchett and Bortel, 1993). The biological basis of this three-dimensional character lies in the inherently asymmetric nature of degeneration in the discs, facet joints, and paraspinal muscles, exhibiting clear convex-concave side differences that both initiate and perpetuate scoliosis progression in a self-sustaining vicious cycle (Geng et al., 2024; Faldini et al., 2013).

Previous reviews on ADS pathogenesis have largely focused on individual structural levels, rarely explaining the mutually driving relationships among coronal, sagittal, and rotational deformities within a unified framework (Kotwal et al., 2011; Petrosyan et al., 2023). Accordingly, this review systematically examines current research progress on ADS three-dimensional pathogenesis along the logical thread of ‘initiation by asymmetric degeneration - amplification by three-dimensional coupling - failure of stabilization systems - modulation by bone quality and genetic background,’ and discusses existing and potential intervention strategies on this mechanistic basis.

## 2 Methods

This review was conducted as a narrative review. Relevant literature was identified by searching the PubMed/MEDLINE database, from inception to July 2026, restricted to publications in the English language. Given the substantial heterogeneity of the evidence bearing on disc, facet joint, paraspinal muscle, and bone quality changes in ADS, and because the aim of this review is to integrate mechanistic findings across these multiple structural levels into a unified pathogenic framework rather than to perform a quantitative synthesis addressing a single, narrowly defined clinical question, a narrative rather than a systematic review approach was adopted. Because this approach is directed at achieving thematic and conceptual saturation across multiple, largely independent evidence domains rather than the recall-maximizing retrieval required for quantitative or systematic synthesis, a single, comprehensively indexed database (PubMed/MEDLINE) was judged an adequate and proportionate search strategy for this purpose; the rationale for this choice, together with its residual limitations, is discussed in Section 11.2.4.

## 3 Asymmetric intervertebral disc and endplate degeneration

### 3.1 Endplate tilting and nucleus pulposus displacement-induced asymmetric loading

The most central pathophysiological event in ADS pathogenesis is the progressive conversion of asymmetric disc and endplate degeneration under axial loading into progressive coronal imbalance (Faldini et al., 2013; Daffner and Vaccaro, 2003). Unlike developmental scoliosis, ADS patients have essentially normal spinal morphology before skeletal maturity, and scoliosis develops *de novo* against a backdrop of degeneration (Aebi, 2005; Daffner and Vaccaro, 2003). This process typically does not begin with a sudden global structural change but rather with localized, asymmetric degeneration of a single disc segment: when slight endplate tilting occurs due to degeneration, the nucleus pulposus shifts within the disc, converting the stress distribution under axial loading from symmetric to asymmetric, with stress concentrating progressively on one side’s annulus fibrosus and endplate margin, establishing the mechanical basis for local wedge deformation (Vergroesen et al., 2015; Walter et al., 2011).

Three-dimensional finite element models of degenerative scoliosis built using finite element methods have provided systematic mechanical evidence for this process. Zheng et al. (Zheng et al., 2015) established and validated the first three-dimensional finite element model for degenerative scoliosis, incorporating asymmetric disc material properties and segmental stiffness parameters to reproduce clinically observed coronal imbalance and segmental range-of-motion changes. Guo et al. (Guo et al., 2025) built upon this foundation to establish and validate a finite element model specific to degenerative lumbar scoliosis, demonstrating high concordance between model-predicted segmental stress distribution and the convex-concave side degenerative differences observed on imaging, providing a methodological basis for subsequent surgical simulation studies.

### 3.2 Axial stress and molecular mechanisms of nucleus pulposus cell degeneration: mechanotransduction and asymmetric stress-specific pathway activation

At the molecular level, recent research has provided relatively direct cellular biological evidence for disc degeneration in ADS. Cai et al. (2023) isolated nucleus pulposus cells from ADS patients with disc degeneration and demonstrated that overloaded axial stress markedly activates the Wnt3a/β-catenin signaling pathway, suppressing expression of extracellular matrix proteins such as type II collagen and aggrecan, upregulating pro-apoptotic proteins including Bax and Caspase-3 while inhibiting Bcl-2, with elderly-derived cells showing significantly greater vulnerability to stress-induced injury than younger cells (Cai et al., 2023).

Critically, however, these molecular mechanisms in the ADS context are not simply the generic intervertebral disc degeneration (IVDD) mechanisms operating symmetrically, but rather appear to represent a mechanotransduction process differentially activated by asymmetric stress. Within an ADS segment, the concave-side nucleus pulposus and annulus fibrosus are subjected to excessive shear force and compressive stress. We speculate that this abnormal mechanosensitive stimulation preferentially activates the mechanosensitive ion channel Piezo1 and the Wnt/β-catenin pathway in concave-side cells, driving upregulation of matrix metalloproteinases and type II collagen degradation, based on evidence that this pathway mediates annulus fibrosus cell apoptosis under mechanical overload (Liu et al., 2023); the convex side, by contrast, is subjected to relatively low or tensile stress and would be expected to show a distinct pattern of apoptotic pathway activation. This differential activation of molecular pathways on the concave versus convex side within the same segment, mediated by asymmetric loading, may help explain the inhomogeneous matrix degradation observed at the molecular level, offering a plausible mechanistic link between the mechanical phenotype (three-dimensional scoliotic deformity) and molecular biological evidence: not simply “disc degeneration leads to scoliosis,” but potentially a causal chain of “asymmetric stress → differential molecular activation → asymmetric matrix degradation → accelerated scoliosis progression,” which remains to be directly validated.

The role of Wnt signaling in nucleus pulposus degeneration has been extended in recent years: Yang et al. (2024) found that matrix protein S100A6 regulates nucleus pulposus cell apoptosis through the Wnt/β-catenin pathway; Zhu et al. (2024) demonstrated that periostin (POSTN) simultaneously activates Wnt/β-catenin and NF-kB pathways to promote nucleus pulposus cell senescence and extracellular matrix metabolic dysregulation; single-cell sequencing studies showed that the Wnt/Ca2+ signaling pathway has significant associations with inflammatory and apoptosis-related gene expression during nucleus pulposus degeneration (Wang et al., 2024). Aberrant activation of the mechanosensitive ion channel Piezo1 can mediate annulus fibrosus cell apoptosis, indicating specific molecular sensors for converting mechanical signals into apoptotic responses (Liu et al., 2023); macrophage polarization state changes play different roles at different stages of disc degeneration (Dou et al., 2025). In the ADS context, these mechanosensitive molecular mechanisms overlap with segmental asymmetric stress to preferentially accelerate degeneration on the concave side bearing abnormally concentrated stress, thereby explaining the lateralized differences in disc degeneration itself at the molecular level.

From a translational standpoint, the Wnt/β-catenin and Piezo1 mechanotransduction pathways described above are, in principle, pharmacologically tractable, raising the possibility of biologic or small-molecule adjuncts that target concave-side matrix degradation directly rather than relying solely on mechanical unloading. However, the mechanistically-based treatment strategies discussed in Section 10 remain confined to biomechanical and structural correction, and do not yet incorporate molecular-level intervention targeting these pathways. Closing this gap between the cellular mechanisms outlined in this section and future adjunct pharmacological strategies represents an important direction for translating the three-dimensional coupling model into clinical practice.

### 3.3 Endplate-disc complex degeneration and cumulative segmental wedging

The endplate serves as the mechanical and metabolic interface between the disc and vertebral body, with its structural integrity critical for maintaining segmental symmetry. Endplate microcracking and osteo-chondral interface damage can significantly impair nutrient diffusion pathways, thereby promoting disc degeneration (Fields et al., 2018); in degenerative scoliosis segments, this endplate injury typically demonstrates clear lateralization, with concave or convex side endplates exhibiting varying degrees of calcification, cystic change, or Modic changes from long-term uneven loading (Costa et al., 2023). This injury pattern has a self-reinforcing character: endplate calcification impairs local nutrient pathways, metabolic demands of nucleus pulposus cells go unmet, matrix synthesis capacity subsequently declines, further aggravating mechanical weakness on that side. As similar asymmetric degeneration accumulates segment by segment in adjacent levels, segmental wedge changes progressively stack, ultimately forming a measurable coronal scoliosis angle (Aebi, 2005; Vergroesen et al., 2015), and providing the structural basis for subsequent coupling of sagittal imbalance and vertebral rotation.

## 4 Coronal-sagittal coupling and vertebral axial rotation

### 4.1 Coupled motion of coronal scoliosis, sagittal imbalance, and axial rotation

The most critical mechanical feature distinguishing ADS from single-plane sagittal deformity is that increasing coronal scoliosis angle is not an isolated event but shows clear correlation with sagittal parameters and vertebral axial rotation, a phenomenon termed ‘coupled motion’ in the biomechanics literature (Costa et al., 2023; Xu et al., 2023). Costa et al. (Costa et al., 2023) systematically summarized three-dimensional coupling mechanisms in scoliosis and their implications for corrective strategy, noting that the coronal, sagittal, and axial rotation planes do not evolve independently but are governed by shared geometric and mechanical constraints, providing a theoretical framework for understanding the three-dimensional features of degenerative scoliosis.

The principal structural and biomechanical features underlying the three-dimensional deformity of ADS are summarized in Figure 1.

**FIGURE 1:**
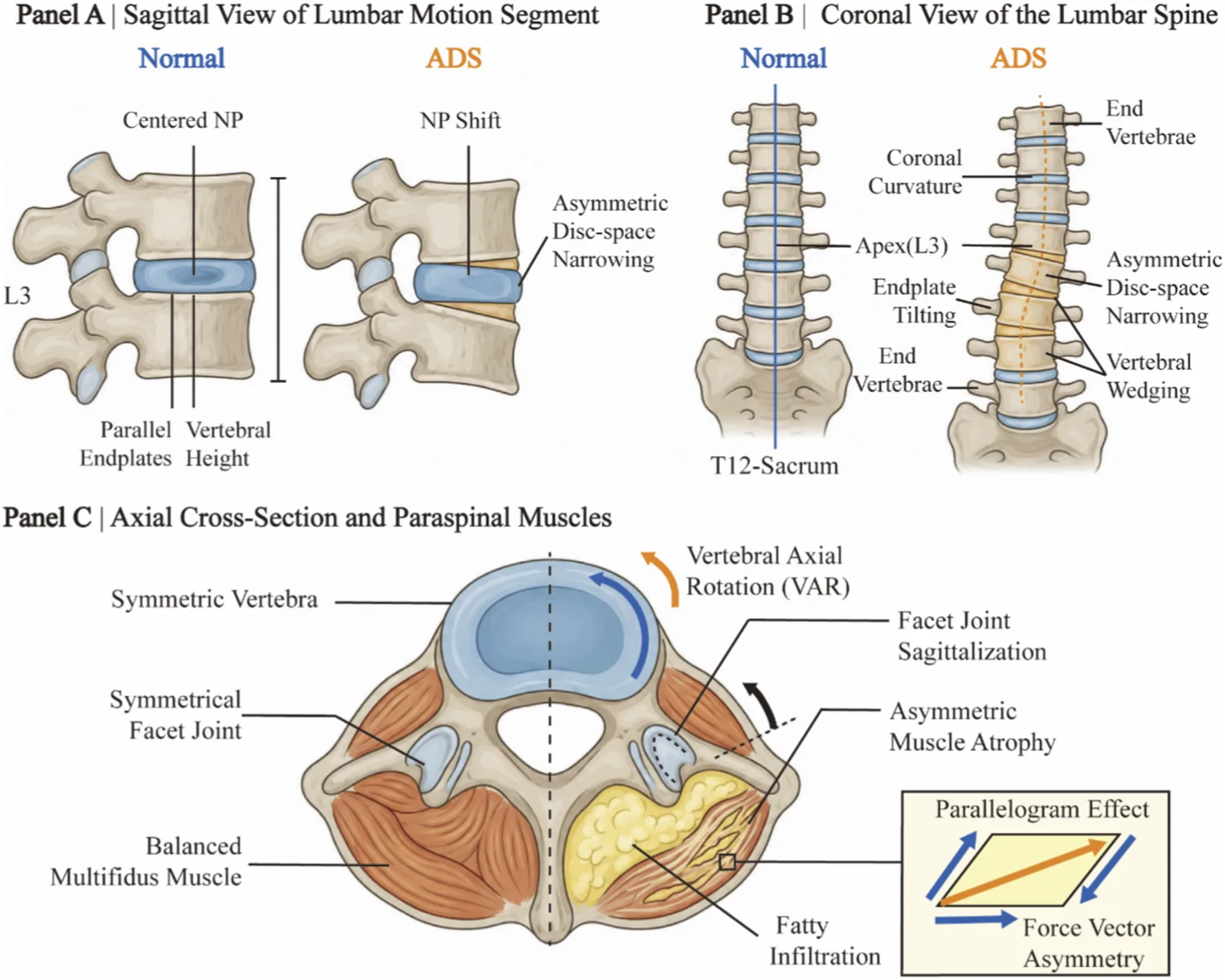
Structural and biomechanical features of adult degenerative scoliosis across the sagittal, coronal, and axial planes. **(A)** Sagittal-plane changes; **(B)** coronal-plane deformity; and **(C)** axial vertebral rotation and paraspinal muscle changes.

Clinical studies using EOS low-dose three-dimensional imaging technology have further validated this coupling phenomenon in degenerative scoliosis populations. Xu et al. (2023) performed three-dimensional analysis of inter-segmental motion in ADS patients undergoing oblique lumbar interbody fusion (OLIF) finding significant correlation between segmental coronal and axial plane motion, and demonstrating that implanting a cage via OLIF to correct coronal scoliosis simultaneously improved axial rotational deformity, providing important clinical evidence confirming the coronal-axial coupling relationship. This retrospective study included 29 consecutive ADS patients (mean age 63.6 years) who underwent staged OLIF surgery between 2018 and 2021, with pre- and postoperative EOS imaging serving as each patient’s own comparator rather than a separate non-surgical control group.

It is worth noting that EOS is one of several tools available for quantifying vertebral axial rotation: conventional plain-radiograph methods (e.g., the Nash-Moe pedicle method, the Perdriolle torsionmeter, and the Raimondi method) and CT-based approaches remain widely used, with differing trade-offs among radiation exposure, reproducibility, and inter-rater reliability compared with three-dimensional EOS reconstruction. Situating EOS within this broader measurement landscape underscores that quantifying axial rotation does not depend on three-dimensional imaging alone, even though dynamic, weight-bearing three-dimensional data remain comparatively scarce (Boyer et al., 2018).

### 4.2 Endplate rotational motion abnormalities and segmental instability

At the segmental kinematic level, *in vivo* endplate motion analysis in degenerative scoliosis patients with different Cobb angles demonstrated that the magnitude of coupled rotation at apical vertebral endplates correlated positively with coronal Cobb angle, and segments with disc degeneration showed higher coupled displacement in lateral bending and rotation, particularly pronounced in the anterior-posterior sagittal direction (Xu et al., 2022). This study recruited 42 DLS patients with the apical disc at L2–L3 or L3–L4, dividing them by coronal Cobb angle into group A (>20°, n = 13) and group B (<20°, n = 28), and further subdividing discs into degenerated (Pfirrmann III–V) and nondegenerated (I–II) groups for within-subject comparison rather than using an external non-scoliosis control group. As disc degeneration deepens and scoliosis angle increases, the structural ‘brakes’ constraining this coupled motion progressively fail, segmental stability continues to decline, and the motion pattern becomes increasingly disordered and propagates segment by segment.

### 4.3 Sagittal imbalance parameters and mutual exacerbation with coronal scoliosis

Sagittal imbalance parameters, including pelvic incidence-lumbar lordosis mismatch (PI-LL mismatch) and increased sagittal vertical axis (SVA), are common comorbidities in ADS patients that closely interact with coronal scoliosis severity. Schwab et al. (Schwab et al., 2013) proposed SVA, pelvic tilt (PT), and PI-LL as the three most critical sagittal parameters predicting health-related quality of life in ASD patients, establishing correction targets (SVA <5 cm, PT < 25°, PI-LL within plus or minus 10°) that provide a quantitative basis for understanding the interaction between sagittal imbalance and coronal scoliosis in ADS (Bauer et al., 2026). In a cohort of 67 ADS patients divided into PI-LL match and mismatch groups based on multifidus fat-infiltration percentage (%FIA) on MRI, PI-LL-mismatched patients showed significantly greater paraspinal muscle degeneration and clinical symptoms compared to PI-LL-matched patients, suggesting that sagittal decompensation may indirectly promote coronal scoliosis worsening by exacerbating paraspinal muscle load asymmetry (Guo et al., 2023).

In a retrospective cohort of 132 ADS patients (no non-scoliosis comparison group), Sun et al. (2019) named the pattern of asymmetric multifidus muscle atrophy, assessed on MRI at the intervertebral levels above and below the apical vertebra, along the diagonal direction of the apical vertebra the ‘parallelogram effect,’ proposing that this diagonally distributed muscle atrophy specifically maintains and worsens sagittal imbalance through mechanics analogous to diagonal support imbalance in a parallelogram. This ‘parallelogram effect’ has direct clinical implications for three-dimensional osteotomy and internal fixation planning: in apical vertebra osteotomy or interbody fusion surgery, surgeons should not only focus on restoration of coronal and sagittal alignment, but should also comprehensively assess the tension imbalance of muscles along the diagonal direction through the apical vertebra. This has potential guidance value for preventing postoperative proximal junctional failure and maintaining long-term balance (Sun et al., 2019). Additionally, when the C7 plumb line deviates toward the convex side of the major curve, L5 tilting is more pronounced, suggesting a bidirectional dependency between coronal and sagittal imbalance (Wu et al., 2025).

## 5 Asymmetric paraspinal muscle degeneration

### 5.1 Imaging evidence of concave-side disuse atrophy and convex-side compensatory changes

Paraspinal muscles, particularly the multifidus, erector spinae, and psoas major, are the core active stabilizing structures maintaining spinal posture. Lee et al. (2025) performed combined CT and MRI analysis of 74 symptomatic ADS patients, systematically comparing convex- and concave-side paraspinal muscle volume and degree of fatty infiltration, finding that concave-side muscle volume of all assessed paraspinal muscles was significantly lower than the convex side, while concave-side fatty infiltration of erector spinae and multifidus was significantly higher than the convex side, and concave-side disc degeneration and neural foramen stenosis were also more severe (Lee et al., 2025). A recent study comparing 145 age- and sex-matched participants (58 controls, 47 conservatively managed ADS, and 40 surgically treated ADS) found that fatty infiltration differences between convex and concave sides exhibit clear heterogeneity across different lumbar segments, suggesting that segment-specific muscle assessment may better reflect local mechanical imbalance than overall measurements (Özcan Ekşi and Ekşi, 2026). An earlier study of 85 patients with degenerative scoliosis found that psoas major and multifidus cross-sectional area at the apical vertebral level was significantly larger on the convex side than the concave side (by 6.3% and 8.4% respectively) (Shafaq et al., 2012).

Beyond cross-sectional CT and MRI, visible asymmetry of the trunk and waistline is a simpler, more accessible clinical sign of this convex-concave muscular imbalance, and validated photographic and surface-topography approaches developed in scoliosis research exist for quantifying such asymmetry, offering a complementary, low-cost, radiation-free means of tracking it alongside cross-sectional imaging. Wider adoption of such methods, however, is constrained by the same lack of methodological standardization discussed in Section 11.2.3.

### 5.2 Neurogenic denervation mechanisms

Recent studies have provided new mechanistic perspectives on paraspinal muscle asymmetry in scoliosis patients from the neuromuscular junction level. Morphological and molecular analysis of paraspinal muscles in scoliosis patients demonstrated that neuromuscular junction-related variables were significantly reduced on the convex side, with denervation marker expression significantly upregulated, indicating neurogenic damage on the convex side, potentially related to neuromuscular junction exhaustion from prolonged high-tension, over-recruitment status on the convex side (Zhang et al., 2025). This finding suggests that convex-side muscles cannot be simply conceptualized as the ‘healthy compensatory side'--though convex-side muscle volume may increase due to compensatory hypertrophy, neurogenic functional damage may simultaneously exist at the innervation level, potentially explaining the progressive exhaustion of convex-side compensatory capacity as the disease progresses.

These distinct muscle pathological phenotypes on the two sides (convex side: neurogenic innervation exhaustion; concave side: disuse fibrosis) are mechanistically significant, and they also provide the direct theoretical basis for developing asymmetric precision physical therapy protocols. For the convex side, intervention should center on neuroprotection and functional restoration; for the concave side, the focus should be on anti-fibrosis therapy and enhanced recruitment--the two-sided intervention strategies being fundamentally different.

### 5.3 Extracellular matrix remodeling

At the molecular level, fatty infiltration and fibrotic changes in concave-side paraspinal muscles have been demonstrated to be associated with activation of specific signaling pathways. Wang Q. et al. (2025) used plasma exosome proteomics to find significantly upregulated cartilage intermediate layer protein 1 (CILP-1) expression in concave-side multifidus of scoliosis patients, accompanied by TGF-beta1/Smad2/3 signaling pathway activation and increased expression of downstream extracellular matrix-related proteins; *in vitro* cellular experiments further confirmed that TGF-beta1 stimulation induces CILP-1 and matrix-related protein upregulation, while Smad3 phosphorylation inhibitors can partially block this effect, suggesting that TGF-beta1/Smad pathway-mediated fibrosis is an important molecular basis of concave-side paraspinal muscle functional decline (Wang Q. et al., 2025). This study enrolled 21 AIS patients (11 undergoing conservative follow-up, 10 requiring surgery) and 20 age- and sex-matched healthy volunteers for plasma exosome proteomic profiling, with findings validated in an independent cohort of 35 AIS patients versus 37 healthy controls by ELISA; concave-versus convex-side paraspinal muscle comparisons were performed within the same surgical patients (n = 5 per side) rather than against an external non-scoliosis control group. Though both convex and concave side muscle degeneration fall within the category of asymmetric paraspinal muscle changes, their molecular mechanisms differ fundamentally: convex-side degeneration is dominated by neurogenic denervation, while concave-side degeneration more prominently manifests as fibrotic remodeling in a low-tension, low-recruitment environment. These two pathways, belonging to different cellular biological routes, jointly result in the overall loss of paraspinal muscle stabilizing function.

### 5.4 Chronic mechanical loading, surgical history, and general muscle catabolic mechanisms

Chronic abnormal mechanical loading is an important driver of muscle degeneration. Sustained mechanical stress can activate FoxO transcription factors and the ubiquitin-proteasome system, upregulating Atrogin-1 and MuRF1 expression to promote muscle protein degradation and fiber atrophy (Bodine and Baehr, 2014); simultaneously, aging skeletal muscle mitochondrial quality control capacity declines, further accelerating atrophy (Romanello and Sandri, 2015). Lumbar surgical history has also been demonstrated to be closely associated with paraspinal muscle health (Song et al., 2023; Suzuki et al., 2022; Han et al., 2023). In the ADS context, the above general degenerative signaling pathways are activated to different degrees in the concave-side low-load disuse environment and convex-side high-load over-recruitment environment, shaping the asymmetric muscle degeneration pattern.

The asymmetric pull and tension imbalance of paraspinal muscles (especially the multifidus) are not merely manifestations of spinal stabilization system failure, but also serve as upstream factors directly influencing the mechanical environment of posterior column facet joints, constituting the critical interface between the active stabilization system and the passive stabilization system, as discussed in the following section.

## 6 Facet joint degeneration and rotational instability

### 6.1 Facet joint orientation asymmetry and the cross-talk between active and passive stabilization systems

Facet joints are important load-bearing and rotation-limiting structures in the posterior column. Three-dimensional anatomical and biomechanical analysis of 240 dry thoracolumbar vertebral columns (Hamann-Todd Osteological Collection) by Masharawi et al. (2004) demonstrated that facet joint orientation varies along the thoracolumbar spine and may show asymmetric or more sagittal configurations. In degenerative lumbar disease, facet tropism has also been associated with vertebral rotation, suggesting that altered facet orientation may contribute to reduced resistance to rotational displacement and abnormal axial loading (Ma et al., 2021). Finite element studies by Park J. S. et al. (2025) and Park et al. (2024) demonstrated that unilateral facet joint degeneration can significantly alter segmental stress distribution and range of motion in axial rotation and lateral bending, causing compensatory load increases in contralateral structures, with instability propagating to adjacent segments. Both studies used a validated L1–S1 finite element model (ROM and disc pressure confirmed against prior FE/*in vitro* data) and compared a normal reference model (G00) against progressively degenerated facet contact grades (G40, G42, G44) under flexion, extension, lateral bending, and axial rotation.

However, facet joint mechanical status does not exist in isolation; it has important bidirectional cross-talk with the paraspinal muscle system. Asymmetric atrophy and tension imbalance of paraspinal muscles (especially the multifidus) directly alters the instantaneous center of rotation (ICR) of the vertebral segment, causing persistent deviation in facet joint loading direction and movement trajectory. Concave-side multifidus traction force decreases due to fibrotic degeneration, weakening posterior column tension support on that side; convex-side multifidus, though compensatorily hypertrophied, has concurrent neurogenic functional damage, also impairing tension control capacity. This asymmetric active pull disrupts the dynamic equilibrium of posterior column facet joints, inducing single-side facet joints to bear abnormal shear forces, thereby driving facet joint orientation toward sagittal remodeling. Conversely, already sagittalized facet joints further lose their constraint on rotation and lateral translation, exacerbating asymmetric stress distribution in paraspinal muscles, organically linking active stabilization system (paraspinal muscles) and passive stabilization system (facet joints, ligaments) degeneration into a mutually amplifying coupling loop (Yu et al., 2025; Kumar et al., 2025). In support of this, a retrospective analysis of 231 chronic low back pain patients (150 with DS, further stratified as mild/moderate/severe, versus 81 non-DS controls) found that multifidus cross-sectional area and functional ratio, together with facet joint angle and overhang length, were independent risk factors for DS on multivariate regression (Yu et al., 2025). A separate cohort of 117 adult spinal deformity patients, stratified by facet joint tropism (side-to-side facet angle difference >10°) versus no tropism, showed that patients with facet tropism had larger Cobb angles, higher pelvic incidence, more advanced facet osteoarthritis and vertebral rotation grades, and smaller concave-side multifidus cross-sectional area (Kumar et al., 2025). Currently, due to the lack of dynamic three-dimensional load imaging technology in clinical practice (existing MRI/CT are supine static imaging), the dynamic evolution of facet joint rotational instability is frequently underestimated in clinical practice--a significant methodological limitation of current research.

### 6.2 Scoliosis as a potential driver of facet joint degeneration

A cross-sectional MRI study of 284 adults proposed a new perspective: scoliosis itself may be an underestimated driver of lumbar facet joint degeneration (Cueva-Medina et al., 2025). The study enrolled 284 non-surgical Ecuadorian adults aged 30–80 years, allocated by Cobb angle into a scoliosis group (n = 142) and a non-scoliosis comparison group (n = 142, Cobb angle <10°); both groups underwent the same anteroposterior lumbar radiography and MRI protocol, as this was an observational study rather than an interventional trial. Within the scoliosis group, participants were further subdivided by Cobb angle into mild (10°–24°), moderate (25°–44°), and severe (≥45°, n = 1, excluded from subgroup analysis due to insufficient power) categories. Using the non-scoliosis group as the reference, multivariate logistic regression found that moderate scoliosis significantly increased the risk of severe facet joint degeneration at all lumbar segments, with odds ratios of 6.72 (95% CI, 2.45–18.39) and 5.57 (95% CI, 2.45–12.67) at L3–L4 and L5–S1, respectively (Cueva-Medina et al., 2025). This finding suggests that the traditional unidirectional causal view may be incomplete; the sustained asymmetric loading caused by scoliosis itself may represent an important contributor to accelerated facet joint degeneration (Ranjbar et al., 2025; Yahara et al., 2023). In a retrospective case–control study of 160 patients undergoing lumbar MRI (2020–2022), participants were allocated by Cobb angle into a scoliosis group (>10°) and a non-scoliosis control group; as an observational study, controls received no intervention and underwent the same MRI assessment. FJT was more prevalent in the scoliosis group than controls (69% vs. 14%) (Ranjbar et al., 2025). Complementing this clinical observation, a biomechanical study combining finite element modeling with surgical specimens from 13 female patients with idiopathic adolescent scoliosis (Lenke type 1, n = 4; type 2, n = 4; type 3, n = 5) demonstrated that over 91% of the mechanical load was transmitted through the facet joints on the concave side, driving subchondral sclerosis and hypertrophy at those joints (Yahara et al., 2023).

### 6.3 Rotational instability and deformity fixation

Following Panjabi (1992) classic theory of the spinal stabilization system, spinal homeostasis is maintained by coordinated functioning of three subsystems: active (muscles), passive (bony and ligamentous structures), and neural control. When capsular and ligamentous passive structures show increased compliance and an expanded neutral zone, the spine more readily undergoes displacement exceeding normal ranges under physiological loading. When both active and passive stabilization structures simultaneously undergo asymmetric degeneration beyond a critical threshold, the three-dimensional deformity gradually transitions from a dynamic state compensable through postural adjustment to an irreversible structurally fixed deformity--the important pathophysiological basis for the clinical progression boundary between ‘mild and compensable’ and ‘severe requiring surgical intervention’ in ADS.

## 7 Bone quality changes and scoliosis progression

### 7.1 Asymmetric bone density loss correlates with scoliosis severity

Bone quality changes are important modulators of ADS progression rate, more reflecting a mutually amplifying relationship between asymmetric loading and asymmetric bone density loss. A retrospective study of 95 degenerative lumbar scoliosis patients demonstrated that, within the same cohort, HU values of vertebral bodies and pedicle screw trajectories around the apical vertebra were significantly lower on the convex than concave side (no separate non-scoliosis control group was included) (Wang et al., 2020). Studies in adolescent idiopathic scoliosis populations showed that with increasing scoliosis severity, convex-side HU value decline was more pronounced while concave-side decline was not significant (Cheng et al., 2022). This was based on a retrospective study of 93 patients with adolescent idiopathic scoliosis in which vertebral body and pedicle screw trajectory Hounsfield unit values were compared between the concave and convex sides of the same curve at multiple periapical levels, without an external non-scoliosis control group. This suggests that the coupling characteristic of ‘greater compressive stress on the convex side, preferential bone mass loss on the convex side’ has important reference value for understanding lateralized distribution of vertebral bone density in ADS patients.

### 7.2 Molecular basis of bone remodeling imbalance

At the bone remodeling level, imbalance in the RANKL-RANK-OPG signaling axis is the core molecular mechanism of bone quality decline in the degenerative context. RANKL upregulation or relative OPG reduction can significantly enhance osteoclast activity, causing cancellous bone mass decline (Boyle et al., 2003); chronic inflammation, estrogen deficiency, and abnormal mechanical loading can all continuously drive bone resorption by modulating the RANKL/OPG ratio (Khosla and Hofbauer, 2017). Osteocytes, as the primary mechanosensory cells within bone tissue, play important regulatory roles in osteoblast differentiation and function through their mechanically-responsive processes (Buck and Stains, 2024). Asymmetric activation of the RANKL/OPG axis is not solely determined by systemic estrogen or age; local asymmetric mechanical stress can differentially regulate osteoclast-osteoblast balance on the convex versus concave sides of the same vertebra through osteocyte mechanosensory function. In the ADS context, this local asymmetric mechanotransduction through the RANKL/OPG pathway may further shift bone remodeling balance on the convex side, making convex-side trabecular structural changes more pronounced, mechanistically echoing the imaging observation of preferential convex-side bone density decline.

### 7.3 Vicious cycle between osteoporosis and scoliosis progression

In the natural history of degenerative scoliosis, a widely recognized vicious cycle exists between osteoporosis and asymmetric loading: asymmetric loading and degeneration jointly trigger and exacerbate scoliosis progression, while postmenopausal osteoporosis, common in women, further amplifies this process, causing weakened vertebrae to undergo progressive asymmetric deformation and collapse under asymmetric stress (Geng et al., 2024; Compston et al., 2019). This vicious cycle not only accelerates coronal scoliosis angle progression, but also increases the risk of vertebral compression fractures and secondary sagittal imbalance, with overall three-dimensional deformity progression rate and severity significantly modulated by the bone quality background.

From a clinical management perspective, this bone-quality-driven amplification loop suggests that interventions directed at the underlying bone quality itself, rather than at the scoliotic deformity alone, may help mitigate ADS progression. Anti-osteoporotic therapies already in standard use for postmenopausal osteoporosis, including antiresorptive agents (e.g., bisphosphonates, denosumab) and anabolic agents (e.g., teriparatide, romosozumab), could in principle slow the asymmetric vertebral collapse that drives this cycle, and preoperative optimization of bone mineral density is increasingly emphasized as part of surgical planning to reduce instrumentation-related complications such as screw loosening and proximal junctional failure. Whether such osteoporosis-directed treatment meaningfully alters the trajectory of coronal or sagittal deformity progression in ADS specifically, as opposed to reducing fracture risk more generally, has not been directly established and remains an important direction for future clinical investigation.

## 8 Genetic background and individual susceptibility

Although the asymmetric degeneration mechanisms at the disc, facet joint, paraspinal muscle, and bone quality levels described above constitute the core pathophysiological basis of ADS, increasing evidence indicates that genetic background plays an important modulatory role in determining individual susceptibility to these degenerative exposures, disease progression rate, and ultimate scoliosis severity.

Genetic studies specifically targeting ADS remain relatively limited; existing evidence derives predominantly from genome-wide association studies (GWAS) in adolescent idiopathic scoliosis (AIS). Multiple GWAS have identified genetic loci associated with AIS susceptibility and severity (Sharma et al., 2011; Khanshour et al., 2018); LBX1, currently the most thoroughly investigated AIS susceptibility gene, may participate in scoliosis susceptibility through influencing somatosensory pathway development (Luo et al., 2020); GWAS in Chinese female patients have also identified novel ethnicity-specific susceptibility loci (Zhu et al., 2015). A large-scale study using the UK Biobank and deep learning technology for precision spine curvature phenotyping identified novel genetic risk loci for scoliosis (Zeosky et al., 2026).

Notably, evidence for extracellular matrix (ECM) gene involvement in spinal degeneration is not confined to AIS: candidate-gene studies in the adult lumbar spine have long identified specific collagen IX variants, such as the COL9A2 Trp2 allele, as being associated with intervertebral disc disease (Annunen et al., 1999), indicating that ECM-related genetic susceptibility is itself an established, if still underexplored, dimension of adult spinal pathology and not merely an extrapolation from pediatric scoliosis genetics.

Building on awareness of this genetic background, the present review further points out an important conceptual gap in existing ADS genetics research: the neglect of epigenetics and gene-environment interaction (G × E) as central considerations in ADS pathogenesis. Elderly populations exhibit significant genetic polymorphisms in susceptibility to sarcopenia and osteoporosis, with genetically determined baseline differences in vulnerability to muscle loss and bone density decline among individuals. When these genetic susceptibility backgrounds encounter the asymmetric mechanical loading environment characteristic of middle and older age, G × E interactions occur: individuals genetically susceptible to sarcopenia or osteoporosis deplete their active stabilization system and bone quality reserves earlier, under equivalent spinal degeneration, and asymmetric degenerative patterns more rapidly progress to decompensated three-dimensional deformities. This G × E framework is the core mechanism explaining why individuals of similar age and degenerative severity exhibit enormous differences in scoliosis progression rate and severity. Future genetic research targeting ADS should prioritize exploring whether sarcopenia- and osteoporosis-related genetic loci (such as VDR polymorphisms and ACTN3 genotype) can predict ADS progression rate; whether epigenetic changes such as DNA methylation and histone modification are differentially activated under asymmetric mechanical stress; and whether environmental modifying factors (such as body mass index and occupational exposure) can amplify G × E effects (Ou-Yang et al., 2023). Intervertebral disc degeneration itself has been demonstrated to have relatively high heritability, with multiple genetic loci related to extracellular matrix regulation closely associated with disc degeneration (Ou-Yang et al., 2023), suggesting that the modulatory role of genetic background may permeate multiple levels of ADS pathogenesis.

## 9 Integrated understanding of the three-dimensional coupling pathogenic model

Synthesizing the evidence across all levels described above, ADS pathogenesis can be conceptualized as a continuous pathological process centered on the disc–facet joint–paraspinal muscle degenerative complex, involving the mutual coupling and potential amplification of three axes: the coronal asymmetric degeneration axis, the sagittal imbalance axis, and the vertebral axial rotation axis. The proposed three-dimensional pathogenic coupling model integrating these interacting mechanisms is illustrated in Figure 2.

**FIGURE 2:**
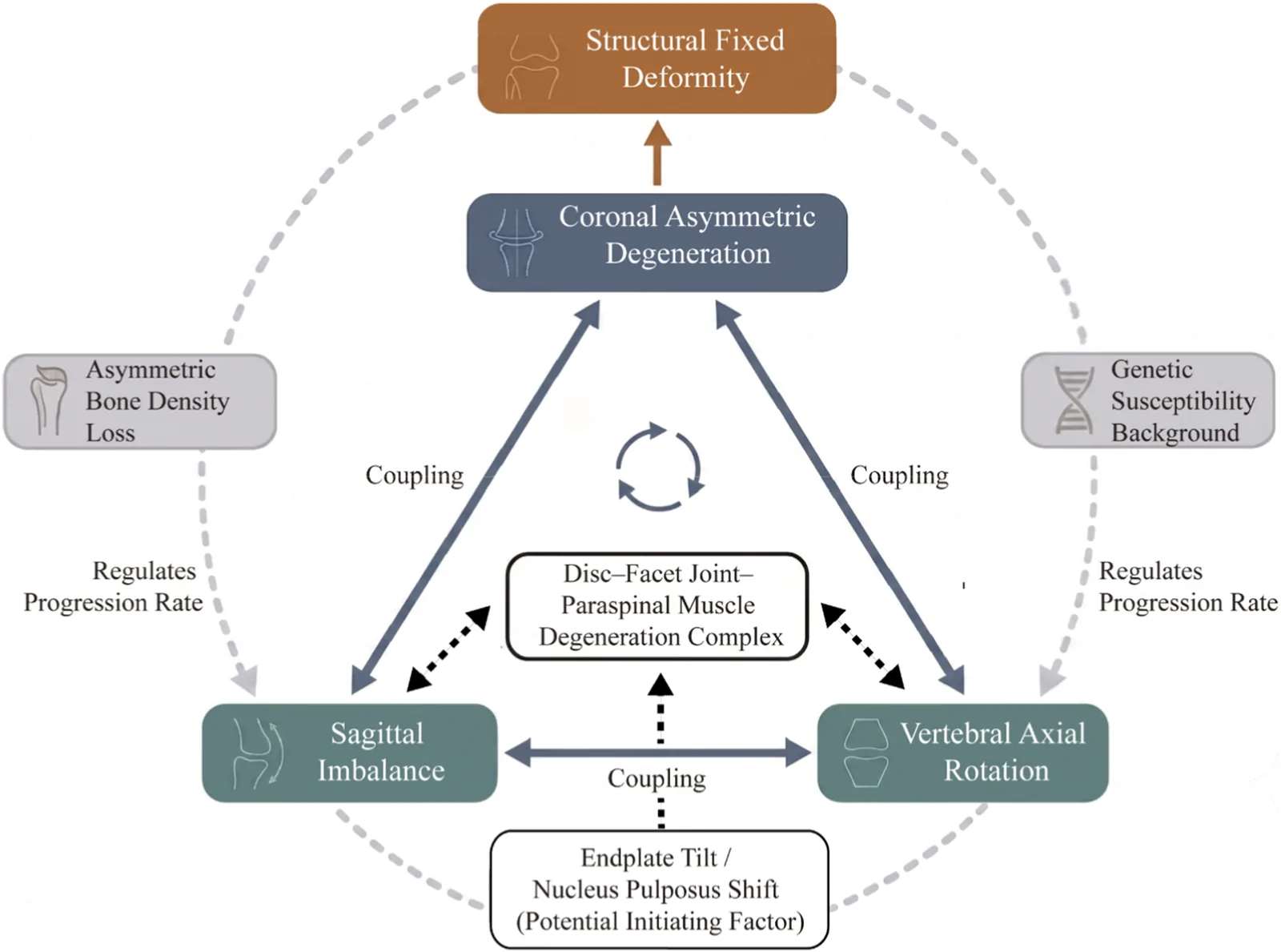
Conceptual model of three-dimensional pathogenic coupling in adult degenerative scoliosis, integrating coronal, sagittal, and axial mechanisms. Solid and dashed arrows indicate relatively direct evidence and hypothesis-generating relationships, respectively.

In ADS, asymmetric degeneration of the discs and endplates may disrupt the bilateral symmetry of segmental mechanical environments. This asymmetry may contribute, through coupled motion, to coronal wedge deformation and vertebral axial rotation; vertebral rotation may further alter loading patterns across the facet joints and ligaments, potentially reinforcing rotational instability. Meanwhile, sagittal imbalance, reflected by increased SVA and PI–LL mismatch, may interact with coronal scoliosis and further modify load distribution between the convex and concave sides of the paraspinal muscles. These mechanical changes may contribute to a transition from relatively symmetric paraspinal muscle morphology toward asymmetric patterns, including convex-side compensatory hypertrophy and concave-side disuse atrophy. Progressive impairment of both active stabilizers (paraspinal muscles) and passive stabilizers (facet joints and ligaments) may, in turn, contribute to the transition from a relatively flexible deformity to a more structurally fixed state. Asymmetric bone quality loss and genetic susceptibility may further modulate the rate and extent of this coupling process over longer time scales, potentially contributing to interindividual differences in scoliosis progression under similar degenerative exposures. The value of this integrative model lies in incorporating previously isolated evidence on disc degeneration, sagittal balance, paraspinal muscle degeneration, and facet joint osteoarthritis into the same three-axis mutually feeding dynamic system, emphasizing that ADS progression is not the linear consequence of any single factor, but a systemic process of multi-plane, multi-structure level degeneration mutually driving and amplifying one another (Petrosyan et al., 2023). This model also provides a conceptual basis for subsequent, mechanism-informed treatment strategies.

It should be emphasized that the Three-Axis Reciprocal Coupling Model is, at this stage, a conceptual synthesis rather than a mechanistically or statistically validated causal system. It is assembled predominantly from cross-sectional imaging correlations, static biomechanical/finite-element simulations, and molecular evidence drawn largely from non-ADS contexts (symmetric intervertebral disc degeneration and adolescent idiopathic scoliosis); direct longitudinal or experimental confirmation that the three axes causally drive one another over time within ADS patients is still lacking. The model is therefore best regarded as a testable framework intended to guide future mechanistic and clinical research, rather than an established explanatory account of ADS pathogenesis.

## 10 Mechanistically-based treatment strategies

### 10.1 Compensatory phase: core and paraspinal muscle function-oriented non-surgical intervention

Non-surgical intervention is appropriate for the early phase when scoliosis angle is small and active-passive stabilization systems can still compensate, with the core objective of delaying asymmetric paraspinal muscle degeneration and maintaining core stabilization function. Existing evidence supports exercise and physical therapy protocols based on core stabilization training combined with side-differentiated correction (such as Schroth therapy), with varying degrees of improvement in Cobb angle, angle of trunk rotation, and quality of life scores, suitable as first-line intervention for patients without neurological compromise or rapid progression signs (Li et al., 2021; Khaledi et al., 2024; Bayram et al., 2024). For example, one randomized-controlled trial enrolled 40 boys with mild AIS (10–18 years), randomized into a Schroth-exercise group (n = 15), a combined Schroth-plus-asymmetric-stabilization group (n = 15), and a waitlist control group (n = 10) that received no intervention during the 12-week trial period (Khaledi et al., 2024). Given the concave-side disuse atrophy and convex-side over-recruitment asymmetric pattern described above, theoretically more targeted strategies should emphasize enhancing concave-side deep stabilizing muscle recruitment while avoiding further convex-side muscle load aggravation. In addition, appropriately dosed mechanical-loading exercise may provide benefits beyond muscle preservation by supporting bone mineral density in older adults, thereby addressing the bone-quality component of ADS pathogenesis. Beyond mechanical and muscle-oriented interventions, biological therapies targeting intervertebral disc degeneration are an active area of investigation, including approaches aimed at modulating matrix metabolism, mechanosensitive signaling, and inflammatory or senescence-related pathways (Miranda et al., 2023). In particular, the Wnt/β-catenin and Piezo1-related mechanotransduction pathways discussed above represent potential biological targets for future disc-directed interventions, although such strategies remain investigational and have not been established as treatments for ADS.

### 10.2 Decompensatory phase: surgical strategies based on three-dimensional correction concepts

Based on coronal-sagittal-rotational coupling mechanisms, modern ADS surgical concepts have evolved from simple coronal Cobb angle correction to three-dimensional simultaneous correction strategies. OLIF approaches insert cages via the convex side, utilizing ligamentotaxis to simultaneously correct coronal wedge deformation and axial rotation while improving sagittal lordosis (Xu et al., 2023); for severe cases with poor flexibility and significant sagittal imbalance, anterior column realignment or three-column osteotomy techniques can further restore overall balance (Park S. J. et al., 2025; Corbett et al., 2024; Zhang et al., 2024). For example, a retrospective study of 210 patients ≥60 years old with severe degenerative sagittal imbalance divided patients into an ACR group and an LLIF-alone group based on the anterior reconstruction technique used; although ACR achieved greater sagittal correction, it carried a significantly higher rate of mechanical failure than LLIF alone (32.3% vs. 14.0%) (Park S. J. et al., 2025). In line with the mechanistic evidence of preferential convex-side bone density loss, some researchers have recommended prioritizing concave-side distraction correction and using larger-size or reinforced anchoring screws around the convex-side apical vertebra to reduce loosening risk (Wang et al., 2020); fusion extent and upper instrumented vertebra selection should also be comprehensively assessed integrating segmental distribution features of facet joint degeneration (Cueva-Medina et al., 2025; Bai et al., 2020).

### 10.3 Interpreting treatment efficacy through the minimal clinically important difference (MCID) framework

A statistically significant improvement in an outcome score does not necessarily indicate a change that is meaningful to the patient. The minimal clinically important difference (MCID) defines the smallest change in a given outcome measure that patients themselves perceive as beneficial, and is increasingly used as the benchmark against which both non-surgical and surgical interventions for ADS should be judged, rather than relying on statistical significance alone.

For the health-related quality-of-life instruments most commonly used to evaluate ADS treatment, anchor- and distribution-based analyses in lumbar spine surgery patients have established MCID thresholds of approximately 12.8 points for the Oswestry Disability Index (ODI), 4.9 points for the SF-36 physical component summary, and 1.2–1.6 points for back and leg pain visual analog scales (Copay et al., 2008). For the SRS-22r, which is central to evaluating both Schroth-based exercise and surgical correction discussed above, MCID values of approximately 0.4 for the total score and 0.2–0.6 across individual domains (appearance, activity, pain) have been reported in patients undergoing surgical correction of adult spinal deformity (Crawford et al., 2015), and subsequent work has confirmed that similar thresholds apply specifically to patients with degenerative lumbar scoliosis rather than only to deformities extending into the thoracic spine (Carreon et al., 2018).

Viewed against these benchmarks, the improvements reported for core-stabilization and Schroth-based exercise protocols in the non-surgical management of ADS (Section 10.1) are frequently of a magnitude that approaches, but does not consistently and clearly exceed, these MCID thresholds, particularly for Cobb angle and SRS-22 domain scores; by contrast, three-dimensional surgical correction more reliably produces changes surpassing MCID for ODI, SRS-22, and pain measures, consistent with the larger effect sizes typically achievable through direct mechanical correction. However, no ADS- or degenerative-lumbar-scoliosis-specific consensus MCID has yet been established for Cobb angle itself, and the thresholds cited above were derived predominantly from adult spinal deformity fusion cohorts and adolescent idiopathic scoliosis populations rather than from the older, comorbidity-laden ADS population addressed in this review; extrapolation of these values to non-surgical, asymmetry-targeted interventions therefore warrants caution. Future studies of ADS-directed interventions, particularly the conservative and biologically targeted therapies discussed in Section 10.1, should report outcomes not only as mean changes with statistical significance but also as the proportion of patients achieving established MCID or substantial clinical benefit thresholds, which would allow more meaningful comparison across intervention types and align with the broader call for methodological standardization raised in Section 11.2.3.

## 11 Discussion

Despite the mechanistic coherence proposed by the Three-Axis Reciprocal Coupling Model, translating these hypotheses into definitive clinical guidance remains restricted by several clear bottlenecks in the current evidence base, spanning biomechanical, molecular, clinical-validation, and methodological domains, as detailed below. Among these, two gaps are the most consequential for the field going forward: the near-total absence of dynamic, three-dimensional biomechanical validation obtained under actual weight-bearing conditions, in contrast to the static supine imaging on which most of the evidence above relies; and the lack of longitudinal, mechanistically instrumented cohorts capable of testing whether asymmetric degeneration at the disc, muscle, and facet joint levels causally drives progression over time, rather than merely correlating with it in cross-sectional data. This section first considers points of genuine conceptual controversy that remain unresolved within the model, before turning to these and other constraints on the underlying evidence base and the conclusions that can be drawn from it.

### 11.1 Controversies in ADS pathogenesis

Controversies exist regarding several conceptual points raised in this review, which remain genuinely contested rather than settled, independent of the evidentiary constraints considered in Section 11.2 below. First, the causal direction between facet joint degeneration and scoliosis is debated: the traditional view holds that facet joint degeneration is a consequence of long-standing coronal deformity, whereas the cross-sectional data discussed in Section 6.2 raise the alternative possibility that scoliosis-related asymmetric loading independently accelerates facet joint degeneration. These two mechanisms are not mutually exclusive and may operate as a bidirectional feedback loop, but which direction predominates at different disease stages has not been established. Second, it remains unresolved whether asymmetric disc/endplate degeneration or asymmetric paraspinal muscle degeneration constitutes the primary initiating event in ADS, or whether both instead arise from a common upstream asymmetric loading state rather than one driving the other; the sequence proposed in this review, with disc and endplate asymmetry as the initiating event, should be regarded as one plausible ordering among several rather than an established temporal hierarchy. Third, the precise threshold separating a compensable, non-surgically manageable deformity from one requiring surgical correction remains a matter of clinical judgment rather than a validated, mechanism-derived criterion; existing surgical indications rely largely on radiographic and symptomatic parameters, and it is not yet established whether the biomechanical stabilization-system markers proposed in this review could meaningfully refine that threshold.

### 11.2 Limitations

To rigorously evaluate the scientific validity of the aforementioned three-axis reciprocal coupling model, it is essential to confront the inherent limitations spanning multiple dimensions of the current evidence base.

#### 11.2.1 Biomechanical and methodological extrapolation constraints

A primary challenge hinges upon the fact that available biomechanical data supporting facet joint and paraspinal muscle instability remain almost entirely static. Supine MRI and CT, by design, cannot capture the complex rotational behavior or dynamic muscle recruitment patterns under actual weight-bearing conditions. Paradoxically, the axial loading that drives asymmetric spinal deformity in the first place is precisely what disappears once a patient lies down for scanning. While finite element methods (FEA) partially compensate for this, they are typically constructed from idealized geometries and boundary conditions calibrated against these same static scans. True, *in vivo* validation against dynamic, three-dimensional load data is still largely absent from the literature. Consequently, we still lack unified animal or *in vitro* models capable of simultaneously reproducing the continuum from early symmetric loading into an irreversible, structurally fixed three-dimensional deformity. Whether the coupling pathways predicted by computers hold up during standing, walking, or trunk rotation requires robust verification, and this methodological gap inevitably limits our confidence in using simulated stress profiles to explain real-world progression mechanics.

#### 11.2.2 Molecular and genetic extrapolation constraints

Furthermore, the molecular and genetic evidence cited throughout this review is rarely ADS-specific. Mechanosensitive pathways such as Piezo1 signaling, along with the matrix degradation cascades described in earlier sections, are drawn predominantly from studies of symmetric intervertebral disc degeneration (IVDD) or adolescent idiopathic scoliosis (AIS). These related but distinct biological contexts fail to capture the asymmetric, senescent microenvironment unique to older adult spines. Extrapolating findings across such diverse phenotypes remains an unproven assumption. Cellular behavior under the specific convergence of aging, chronic asymmetric mechanical stress, and pre-existing degenerative disease may differ in ways that current cross-extrapolated data simply cannot resolve. Dedicated molecular studies using paired, patient-derived tissue that specifically compares the concave and convex sides within the same asymmetric microenvironment are practically non-existent.

#### 11.2.3 Clinical validation and methodological standardization constraints

Finally, this lack of mechanistic standardization directly compromises the validation of clinical interventions. Existing non-surgical studies are frequently underpowered, lacking high-quality randomized controlled trials (RCTs) stratified by specific asymmetric degeneration phenotypes. This translational friction is further compounded by a profound lack of methodological uniformity in clinical imaging; paraspinal muscle fatty infiltration scoring remains poorly standardized (Han et al., 2021; Hodges et al., 2021), and definitions for facet joint articular asymmetry vary widely across protocols (Gao et al., 2017). The same lack of standardization extends beyond static imaging to non-invasive spinal curvature assessment tools themselves: surface topography, rasterstereography, and other radiation-free posture- and kinematics-based systems show substantial inter-device and inter-protocol variability, with reliability and criterion validity still inconsistently established across platforms (Mehta et al., 2023; Padulo and Ardigò, 2013). Until these methodological and biological links accumulate dedicated confirmation, several of the core systemic pathways proposed in this model should be critically regarded as biologically plausible hypotheses rather than confirmed certainties.

#### 11.2.4 Search methodology limitations

This review relied on a single database, PubMed/MEDLINE, restricted to English-language publications, a choice that warrants explicit justification rather than being treated only as a shortcoming. Multi-database searching is conventionally required for systematic reviews and meta-analyses because omitting even a small proportion of studies can materially shift a pooled quantitative estimate (Bramer et al., 2017), whereas this review does not pool effect sizes but instead integrates mechanistic evidence from five largely independent structural domains into a conceptual, hypothesis-generating framework (Section 9) whose coherence rests on convergence across multiple, mutually corroborating studies rather than on any single database-specific finding; accordingly, methodological guidance for narrative reviews (e.g., SANRA) does not require multi-database searching or PRISMA-style documentation (Baethge et al., 2019), and a dedicated evaluation of orthopedic meta-analyses found that MEDLINE alone achieved 90% recall against the gold-standard set of included studies, only marginally below the 91% achieved by combining MEDLINE and EMBASE, with Embase’s incremental yield concentrated mainly in pharmacological and grey/non-English literature of limited relevance here (Slobogean et al., 2009). Nonetheless, this strategy carries residual limitations: mechanistic studies indexed only in other databases (e.g., Embase, Scopus, Web of Science) or published in other languages may have been missed; the review was not prospectively registered, and no formal risk-of-bias or quality-appraisal tool was applied, with selection and synthesis instead relying on a qualitative, thematically organized narrative approach more susceptible to selection and interpretation bias than a fully systematic methodology; and because the search was conducted as of a single date, this review is a cross-sectional rather than a living, continuously updated synthesis, and its conclusions should be interpreted with these procedural boundaries in mind.

## 12 Conclusion and future perspectives

Research on adult degenerative scoliosis pathogenesis has made remarkable advances over the past decade, progressing from initial descriptive recognition focused on disc and facet joint degeneration to increasingly refined levels including asymmetric molecular mechanisms, paraspinal muscle neurogenic damage, lateralized bone density distribution, and coronal-sagittal-rotational three-axis coupling. The Three-Dimensional Coupling Pathogenic Model proposed in this review attempts to integrate these dispersed mechanistic evidence into a unified systematic framework, emphasizing that the essence of ADS is a dynamic process of asymmetric degeneration mutually driving and collectively amplifying across three spatial planes. This perspective differs from the traditional single-plane sagittal decompensation mechanism framework and more accurately reflects the pathophysiological characteristics of ADS as a genuine three-dimensional deformity.

Future research should bridge foundational cellular mechanisms with translational clinical applications. At the mechanistic level, there is an urgent need to establish specialized animal models and single-cell or spatial multi-omics technologies that faithfully map the heterogeneous, senescence-associated microenvironments (such as mechanosensitive Piezo1 activation and Wnt/β-catenin signaling dynamics) of concave-versus convex-side cells under chronic asymmetric stress. On the translational side, these cellular and molecular insights open new avenues for mechanism-informed interventions. For instance, elucidating concave-side fibrotic pathways and convex-side neurogenic denervation may eventually inspire targeted pharmacological adjuncts or side-specific rehabilitation strategies; similarly, understanding local RANKL/OPG imbalance can refine anti-resorptive protocols in osteoporotic ADS patients. However, transitioning these hypotheses into clinical practice requires rigorous validation through mechanistically stratified, high-quality prospective clinical trials, moving beyond empirical management toward mechanism-driven precision medicine.

Particularly promising is the potential for artificial intelligence and multimodal data integration technologies to bring meaningful opportunities to ADS research. The core future direction lies in using deep learning frameworks to panoramically integrate patients’ genetic susceptibility target data (SNP arrays or whole-genome sequencing), dynamic three-dimensional biomechanical finite element model data, and paraspinal muscle radiomics features derived from MRI/CT, constructing precision models capable of predicting individualized ADS progression rate and trajectory. Building on this foundation, further integration with intraoperative three-dimensional navigation data and postoperative functional outcome feedback holds promise for establishing intelligent assisted decision-making systems for individualized ADS surgical planning and prognosis prediction. This multimodal AI integration pathway will be the core technological support for driving ADS from ‘empirical’ clinical practice to ‘mechanism-driven precision medicine (Wang D. et al., 2025; Hiyama et al., 2025).

As three-dimensional imaging technology, biomechanical modeling, and multi-omics analytical methods continue to advance, deepening the understanding of ADS three-dimensional pathogenesis from a systemic integrated perspective will provide an increasingly solid theoretical foundation for early identification, precision intervention, and surgical decision-making optimization for this increasingly prevalent spinal disease of the elderly.
